# The Effect of Platelet Rich Plasma on Chondrogenic Differentiation of Human Adipose Derived Stem Cells in Transwell Culture

**Published:** 2013-11

**Authors:** Mohammad Mardani, Azadeh Kabiri, Ebrahim Esfandiari, Abolghasem Esmaeili, Abbasali Pourazar, Malekmassoud Ansar, Batool Hashemibeni

**Affiliations:** 1 Department of Anatomical Sciences and Molecular Biology, Faculty of Medicine, Isfahan University of Medical Sciences, Isfahan, Iran; 2 Department of Anatomical Sciences, Paramedical school, Guilan University of Medical Sciences, Langeroud, Iran; 3 Cell, Molecular and Developmental Biology Division, Department of Biology, Faculty of Sciences, University of Isfahan, Isfahan, Iran; 4 Department of Immunology, Faculty of Medicine, Isfahan University of Medical Sciences, Isfahan Iran; 5 Department of Anatomical Sciences, Faculty of Medicine, Guilan University of Medical Sciences, Rasht, Iran

**Keywords:** Adipose-derived stem cells, Platelet-rich plasma, Transwell culture

## Abstract

***Objective(s):*** Platelet-rich plasma (PRP) has recently emerged as a promising strategy in regenerative medicine due to its multiple endogenous growth factors. Little is known about the role of PRP as a promoter in chondrogenesis of human adipose derived stem cells (hADSCs). The aim of this study was to determine whether PRP may be considered as a natural and easy achievable source of growth factors to promote the chondrogenic differentiation of hADSCs in Transwell culture.

***Materials and Methods*** *:* Biochemical, immunohistological and molecular assays were used to evaluate the effect of different concentrations (5%, 10%, and 15%) of PRP on chondrogenic differentiation of hADSCs in Transwell culture.

***Results*** *:* The cells in the presence of 10% PRP produced markedly higher amounts of GAG and DNA, in comparison to the control group. PRP also increased chondrogenic markers in these cells, such as sox-9, aggrecan and collagen type II. A high expression level of collagen type X as a hypertrophic marker was observed in cartilage produced by using either PRP or TGF-β1.

***Conclusion*** *:* Our findings indicate that autologous PRP at an optimum concentration had beneficial effects on differentiation of hADSCs in Transwell culture. Further, *in vivo* studies are necessary to fully define the clinical implications of PRP.

## Introduction

Articular cartilage is an avascular tissue with low cellular biosynthetic activity resulting in a limited capacity for self-repair (1, 2). Therefore, tissue engineering has been considered as a promising alternative for the cartilage repair. This strategy requires several key elements cells with high chondrogenic potential, chondrogenic growth factors and a 3-dimensional scaffold (3). Research focusing on cartilage repair is currently shifting towards utilization of mesenchymal stem cells (MSCs). MSCs (derived from bone marrow, periosteum, synovium, muscle and adipose tissue) have the potential to differentiate into various tissues including cartilage and their capacity for self-renewal is significantly considerable (4). Compared to other sources of mesenchymal cells, adipose tissue has the advantage of being readily available from patients in relatively large quantities (5, 6). Adipose derived stem cells (ADSCs) keep their multipotency *in vitro* for several culture passages  (7). The critical issue for the application of ADSCs in cartilage tissue engineering is determining the appropriate conditions to control cellular differentiation. It has been shown that chondrogenesis in ADSCs can be enhanced in 3D culture (8) and in the presence of growth factors; such as transforming growth factor-β (TGF-β) (9), insulin-like growth factor 1 and bone morphogenic protein 6 (BMP-6)  (10). However, the complexities involved in the safety and efficacy of either exogenous or genetically induced growth factor deliveries have led investigators to examine other mechanisms for inducing chondrogenesis (11).

Platelet-rich plasma (PRP), an autologous derivative of whole blood, has been used in clinics for few decades. PRP is rich in growth factors including TGF-β, Insulin-like growth factor (IGF), platelet-derived growth factor (PDGF), basic fibroblast growth factor (bFGF) and vascular endothelial growth factor (VEGF) (12, 13). The growth factors associated with PRP have an important role in soft and hard tissue repair (14). The importance behind such a use refers to the great quantity of growth factors present in a well-prepared PRP concentrate (15). PRP preparations are versatile and reduce medical costs (16). PRP derived growth factors can effectively induce human nucleus pulposus (hNP) proliferation and differentiation      (17). It has been reported that PRP stimulates porcine chondrocyte proliferation and matrix biosynthesis (18). Furthermore, some *in vivo* studies have shown that chondrocyte/PRP composites could enhance the regeneration of articular cartilage defects (19).

For chondrogenesis ADSCs need to acquire a rounded cell shape. This can be achieved via two distinct procedures, using a scaffold free system such as pellet culture or Transwell culture, or from applying scaffolds such as alginate, and agar (20). Human bone marrow stem cells (hBMSCs) have been shown to undergo chondrogenic differentiation in Transwell cultures (21). The current study therefore aims to investigate chondrogenic effects of PRP on hADSCs in a Transwell culture system.

## Materials and Methods

***Isolation and culture of hADSCs***

Human subcutaneous adipose tissue was obtained by elective surgery from three female subjects. All procedures were carried out according to the Isfahan University of Medical Sciences, Medical Faculty Ethics Committee Approval. Adipose tissue was washed thoroughly with sterile PBS (phosphate buffer saline) (Sigma), finely diced and then digested with 0.1% collagenase A (Sigma) solution for 30-45 min at 37°C. The enzyme was neutralized with an equal volume of expansion medium containing DMEM -low glucose (Sigma) plus 10% FBS (Invitrogen) and 1% penicillin/streptomycin (Gibco). Following this, the suspension was centrifuged, and the cell pellets were placed on culture flasks containing fresh expansion medium. The following day, the medium was changed and thereafter every 3-4 days. After reaching desired confluency, cells were trypsinized with trypsin –EDTA (Invitrogen) and were counted using a hemocytometer. The ADSCs were then plated onto plastic tissue culture dishes at a density of 8000 cells /cm^2^. After three passages, cells were used for the following experiments.

***Characterization of ADSCs***

In order to determine superficial markers of expanded ADSCs, the cells were harvested at passage three by trypsinization. After two washes with PBS, they were incubated with monoclonal antibodies against CD90, CD3, CD19 (IQ product), CD14, and CD45 (LeocuGATE), CD44 (Dako), CD105 (Abcam) for 30 min. Primary antibodies were directly conjugated with fluorescein isothiocyanate (FITC) or phycoerythrin (PE). Nonspecific FITC or PE- conjugated IgG were used as an isotype control. The cells were analyzed using BD FACSCalibur machine (BD Bioscience) and WinMDI software.

***Preparation and activation of PRP***

Allogenic human PRP was obtained from Isfahan, Alzahra Blood Bank. PRP from a total of 5 donors was mixed to achieve representative values of growth factors. The final volume of mixed PRP (10 ml) was then activated by 1000 µl thrombin activators (1000 unit bovine thrombin (Sigma) in 1000 µl 10% calcium chloride). The mixture was allowed to undergo the maximal clot retraction at 4°C overnight, and was centrifuged at 3000 g for 10 min. The supernatant (hPRP released growth factors) was collected and stored at -80°C.

***Measurements of the levels of TGF-β1 in activated PRP***

The levels of TGF-β1 were measured using a DuoSet ELISA Development System (DY240, R&D). In this assay, a dilution series of TGF-β1 standards was prepared in 100 µl volumes in 96 well microtiter plates coated with TGF-β1 receptor II. The TGF-β1 in the sample was converted to the activated form using an acidification/neutralization process. The TGF-β1 concentration was determined from standard curve prepared from seven TGF-β1 standard dilutions. Each sample and TGF-β1 standard dilutions were done in duplicate.

***Chondrogenic differentiation***

Harvested hADSCs from passage 3, were resuspended in chondrogenic culture medium (high glucose Dulbeco’s modified Eagle medium (Gibco), supplemented with 100 µg sodium pyruvate (Sigma), 10 ng /ml TGF-ß1 (R&D Systems), 100 nM dexamethasone (Sigma), 1% ITS +Premix (Sigma), 40 µg/ml proline (Sigma), 50 µg/ml ascorbate -2-phosphate (Sigma) and 1% penicillin- streptomycin (Gibco)) (22). For chondrogenesis in Transwells, 

h ADSCs aliquots (5 × 10 ^5^ in 100 µl of medium) were pipetted onto dry filter inserts of Transwells in multiwall plate (6.5 mm diameter 0.4 -µm pore size polycarbonate membrane, 24 well plates (Corning Life Sciences)). The plate was centrifuged at 200 g for 5 min. Then 0.5 ml of chondrogenic medium was added to the lower chamber. The medium was replaced every 2 days. This was also carried out for the medium in the upper chamber. In the control group, incomplete chondrogenic medium was used containing no TGF-ß1. To evaluate the effect of PRP in chondrogenesis, we applied PRP in three different concentrations (5%, 10% and 15%) to replace the TGF- β1 in the chondrogenic medium. All other conditions and components of the chondrogenic medium remained the same. The cells were kept in chondrogenic medium up to two weeks. 

***Biochemical analysis***

Samples were digested with Sigma papain digestion solution (125 µg/ml) for 16 hr at 60°C. Double- stranded DNA (dsDNA) content was determined with Hoechst 33258 (Sigma) using calf thymus DNA as standard. The sulfated glycosaminoglycan (sGAG) concentration was measured with the dimethylmethylene blue (Sigma) using bovine trachea chondroitin sulfate as a standard.

***Immunohistochemistry***

Immunohistochemistry was performed on specimens from day 14. All samples were fixed in 10% formalin overnight and then embedded in paraffin and sectioned at 5 μm. Antigen retrieval for collagen II was performed through incubation with 8 mg/ml hyaluronidase (Sigma) for 2 hr at 37°C, whereas antigen retrieval for collagen X required 2 mg/ml hyaluronidase (Sigma) for 1 hr. In addition, collagen X samples were treated with 1 mg/ml Pronase (Sigma). Nonspecific binding sites were blocked with blocking buffer, and sections were incubated overnight with primary antibodies at 4^o^C. Monoclonal antibodies directed against human antigens were available for type II collagen (ab3092; Abcam) or type X collagen (C7974; Sigma). Sections were washed and incubated with anti-mouse IgG secondary antibody (ab2891; Abcam) that was linked to horseradish peroxidase and was developed using 3, 3^′^- diaminobenzidine (DAB) substrate kit (ab94656; Abcam). Human articular cartilage and osteochondral plugs were prepared in the same manner as positive controls of collagen type II and collagen type X respectively.

***RNA isolation and quantitative RT-PCR***

RNA samples (n = 3 per each group) were prepared after 14 days of chondrogenic differentiation. Discs were disrupted in liquid nitrogen using a small pestle and then RNA was isolated using TRIzol reagents (Invitrogen). The RNA was reverse transcribed using RevertAid First Strand cDNA Synthesis Kit (Fermentase) with oligo dT primers. The real-time polymerase chain reaction was performed using SYBRGreen PCR Master Mix (Fermentase) and the StepOne Plus™ quantitative real time PCR detection system (Applied Biosystems). Primers were designed for each gene using the AlleleID software (Primer Biosoft), which generated the following sequences: collagen II (forward-CTGGTGATGATGGTGAAG, reverse- CCTGGATAACCTC TGTGA), collagen X (forward-AGAATCCATCTGAGAATATGC, reverse-CCTCTTACTGCTATACCTTTAC),sox-9(forward-TTCAGCAGCCAATAAGTG,reverse-GTGGAATGTCTTGAAGGTTA), aggrecan (forward-GTGGGACTGAAGTTCTTG, reverse-GTTGTCATGGTCTGAAGTT) and GAPDH (forward-AAGCTCATTTCCTGGTATG reverse-CTTCTTCTTGTGCTCTTG). The gene of interest was normalized against the reference gene glyceraldehyde-3-phosphate dehydrogenase (GAPDH). The expression level of each target gene was calculated as 2^-∆∆Ct^, as previously described (23, 24).

**Figure 1:**
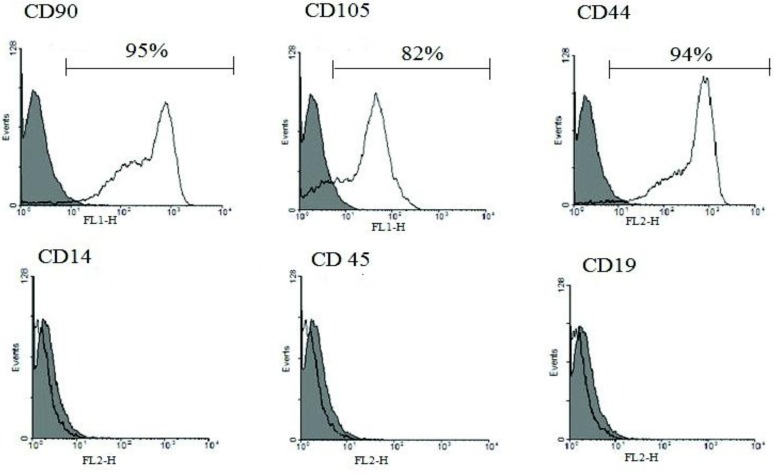
Characterization of human ADSCs. Flow cytometric analysis of human ADSCs using specific FITC and PE coupled antibodies against surface markers. An isotype control is included in each test (Gray lines). Flow cytometric analysis show that human ADSCs express CD90, CD44, and CD105 markers but do not express CD14, CD45, and CD19

***Statistical analaysis***

The comparison of GAG assay, DNA assay and gene expresion between groups, was carried out with one-way ANOVA analyse of variance and *Post hoc* (Tukey) analysis. Those with *P-value *of < 0.05 were considered statistically significant. All data were reported as mean ± SE.

## Results

***Flow cytometry results***

Human ADSCs were characterized in respect to the expression of surface antigen. In our study 95±5%, 83±6% and 94±5% of ADSCs expressed MSC specific markers, including CD90 (Thy-1), CD105 (Endogelin) and CD44 (Hyaloronate receptor), respectively. Meanwhile FACS analysis of ADSCs showed low levels of CD45 (Leucocyte common antigen), CD14 (Monocyte differentiation antigen) and CD19 (B-lymphocyte antigen) (Figure 1).

***Platelet counts and level of growth factor***

The platelet counts in hPRP preparations were 10.16 × 10^6^/ml for a total of 5 donors. The concentration of TGF-ß1 in PRP was 277 ng/ml.

***Biochemical analysis***

**Figure 2:**
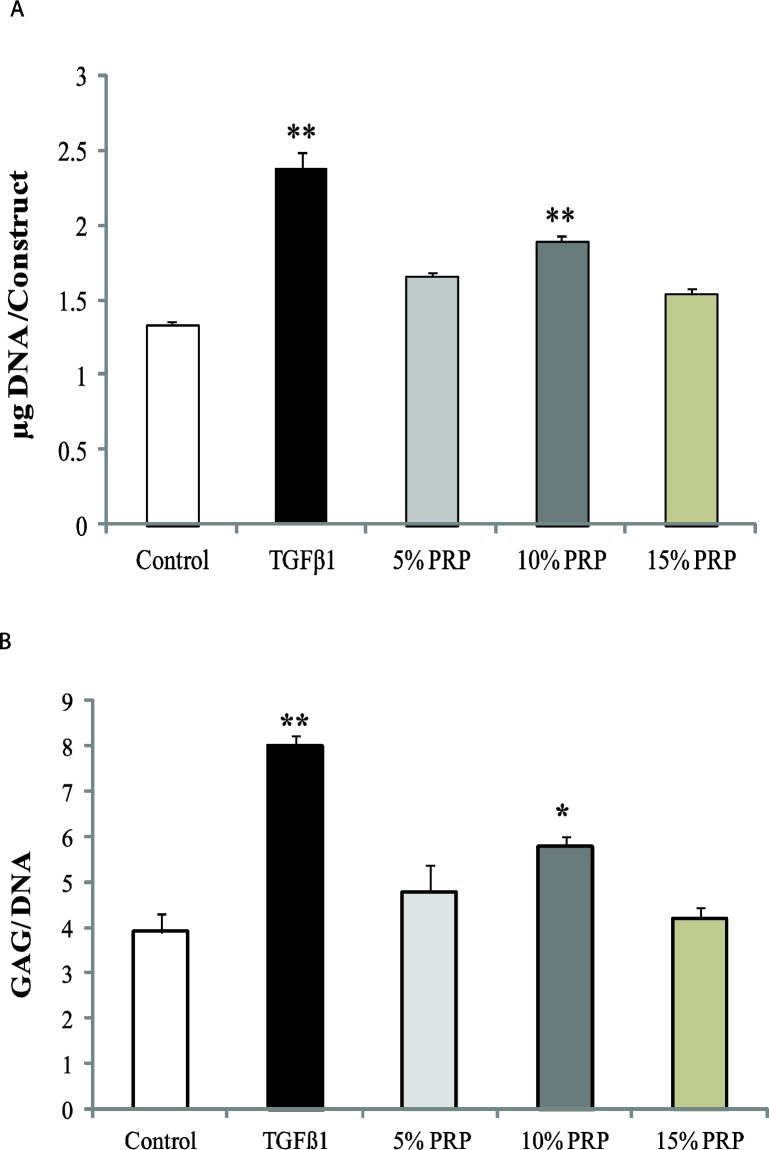
Biochemical analysis of each construct. A) Total DNA (µg) per construct in different groups. B) Glycosaminoglycan (GAG) content per DNA in different groups. Asterisk indicates that the medium condition is significantly different from control by ANOVA (*(*P*<0.05), ** (*P*<0.001)). Error bars represent standard error of the mean

Cell proliferation was measured by using dsDNA as a surrogate and was expressed as the amount of each constructs DNA after 14 days (Figure 2, A). The amount of sulfated GAG was evaluated using DMMB assay and was presented in terms of GAG per DNA (Figure 2, B). All values of different groups were compared to the control group. In both assays, PRP increased DNA and GAG amounts. PRP acted as an inducer of proliferation and at the same time it fostered the production of sulfated GAG in discs in comparison to control group. The highest proliferative response was observed in TGF-β1 group (2.38 ± 0.11) (*P< *0.001) and then in 10% PRP (1.9±0.03) (*P<*0.001). This amount was less in 15% PRP group. In the 5% PRP group it was 1.**7**±0.03. The same trend was observed in the GAG/DNA groups. The TGF-β1 induced highest GAG/DNA ratio in discs (8±0.2) (*P<*0.001). This was 5.8±0.2 (*P<*0.05) for 10% PRP group and was 4.8±0.6 for 5% PRP group.

***Immunohistochemistry***

**Figure 3:**
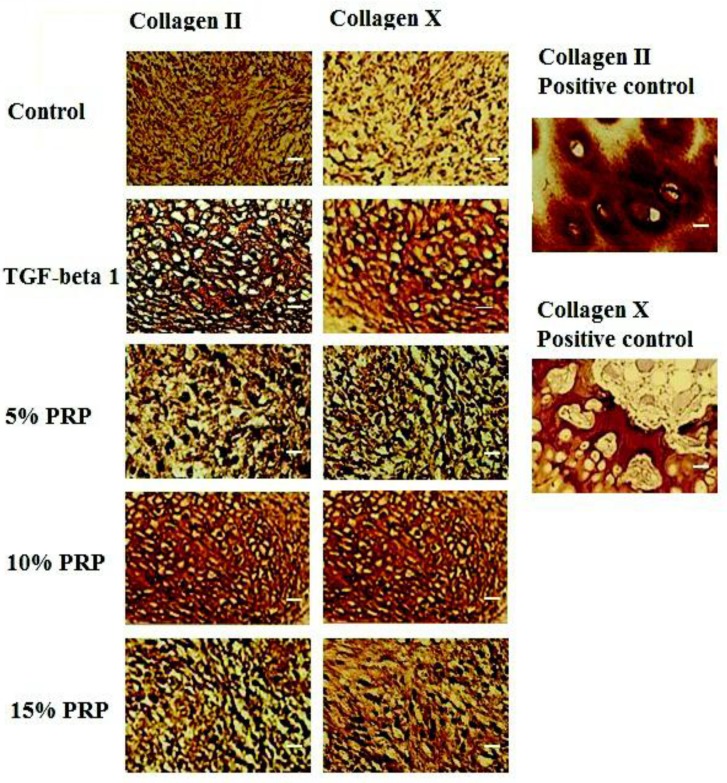
Immunohistochemistry staining. First column shows the staining for type II collagen in control, TGF-β1, 5% PRP, 10 % PRP and 15 % PRP groups. Second column represents immunohistochemistry staining of different groups’ samples for type X collagen. Third column: positive control samples. Upper insert shows the human articular cartilage as a control for type II collagen and lower insert represents the human osteochondral plug as control for type X collagen. Scale bar: 20 µm

Significant deposition and accumulation of ECM components within the discs were evident through immunohistochemical analysis, specifically in type II collagen, the primary collagen found in articular cartilage. Type II Collagen was uniformly distributed in the TGF-ß1 group and in the 10 % PRP group (Figure.3) but in the other groups, type II collagen distribution was less homogenous (Figure 3). Type X collagen, a cartilage hypertrophic phenotypic marker, was observed within the collagenous matrix of all groups. The distribution pattern of type X collagen was the same as type II collagen (Figure 3). 

Specimens were processed using identical protocols, while the human articular cartilage and human osteochondral plugs were used as the positive controls.

**Figure 4:**
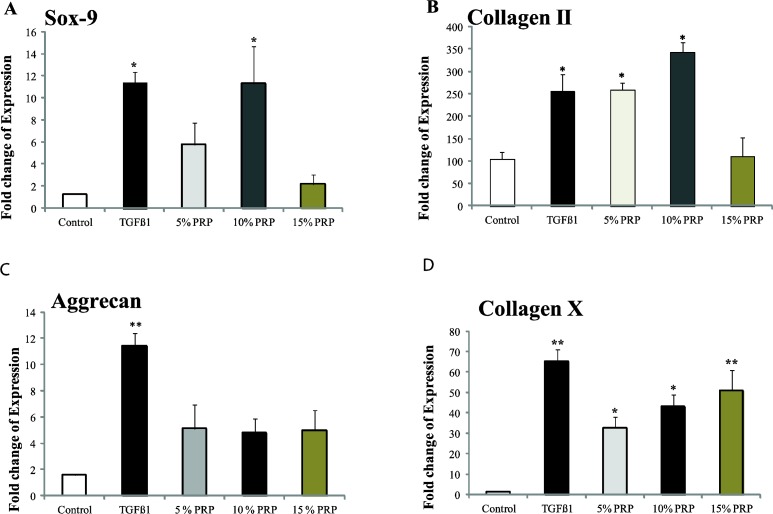
Day 14 reverse transcriptase-polymerase chain reaction for different groups. Data presented as fold changes from day 0 monolayer cells A) Sox-9, B) Collagen type II, C) Aggrecan and D) Collagen type X. Error bars represent standard error of the mean. Asterisk indicates that the medium condition is significantly different from control by ANOVA (*(P<0.05), ** *(P*<0.001*)*)

***Quantitative RT- PCR***

The general patterns of gene expression are summarized in Figure 4. GAPDH was chosen as the reference gene. The mRNA level of SOX-9, collagen type II and aggrecan (three positive markers for chondrogenesis) increased significantly in TGF-ß1 group as compared to the control group. The level of SOX-9 mRNA in 10% PRP group was markedly increased. The mRNA level of collagen type II enhanced significantly in 5% and 10% PRP groups. However, in the cultures containing PRP, the level of aggrecan mRNA was similar to the control. No significant enhancement of these genes was observed in 15% PRP group.

The mRNA expression of collagen type X (a negative marker for chondrogenesis) was increased significantly in all groups. The highest expression level was observed in TGF-β1 group (65-fold) as compared to the control group *(P<*0.001*)*. 

## Discussion

Although PRP has been used in clinical research for decades, little is known about the effects of *in vitro* PRP on the chondrogenesis of ADSCs. In this study, we have evaluated the capability of PRPs in inducing differentiation of human ADSCs toward chondrogenic lineage in Transwell culture. 

Our findings revealed that the growth factors derived from platelets can promote cell proliferation, increase the extracellular matrix (ECM) synthesis as well as up-regulate the expression of chondrogenesis-specific markers. Growth factors are important for tissue repair, especially in the cartilage regenerative process (25). Due to the problems involved in the safety and efficiency of recombinant growth factor, there is a limitation for exogenous growth factors to be applied in clinical treatments (26). In contrast, PRP is a natural cocktail of growth factors that is easily obtained from peripheral blood. Therefore, use of prepared PRP from a patient’s own blood will possibly decrease harmful immune responses  (27). Meanwhile, preparing PRP is not expensive in comparison to preparing exogenous growth factor.

It has been reported that PRP could stimulate the rapid growth of ADSCs and also promote proliferation of human periodontal ligament and mesenchymal stem cells (-). PRP can enhance adhesion, proliferation and differentiation of human periodontal ligament cells (hPDLC) (28). PRP also plays remarkable roles as a storage vehicle of growth factors including TGF-β, IGF, and PDGF (18, 27).

TGF-β induces the MSC proliferation and the accumulation of ECM (31). IGF can stimulate cell proliferation, and has an additive effect when mixed with TGF-β and stimulates ECM synthesis. PDGF can also increase ECM synthesis (32). Platelet lysate (PL) is an appropriate substitute for fetal calf serum in expansion medium of MSCs and causes reduction in the time required to reach confluence  (33). 

In the current study, we used different concentrations of PRP (5%, 10 % and 15%) in chondrogenic media. To avoid the problems of the differences in PRP preparation and to compare the results of this study with other studies, the concentration of TGF-β1, the main growth factor of platelets, quantitatively was tested using ELISA assay. 

The findings of our study revealed that the effect of PRP is dose dependent and 10 % PRP seemed to be the optimum concentration. This is in agreement with a previous study showing that 10% buffered PRP in chondrogenic media, is an optimal concentration (29). 

In the present study, it was shown that there is an up-regulation in the gene expression profile of sox-9, collagen type II, aggrecan and collagen type X in response to PRP. This suggests that TGF-ß1 produced by PRP (at 10% concentration) was capable of acting as a chondrogenic inducer. The expression profile for these genes was very similar to that of the recombinant TGF-ß1 group.

PRP have been used in different forms, such as clot and fibrin sealant that this may lead to a reduction in collagen type II gene expression (34). In our experiments PRP was added to the components of the incomplete chondrogenic media as a source of growth factor. It was also observed that there is an enhancement in the expression of Sox-9 and aggrecan in hADSCs, which was in consistent with other studies (29). In our study, high level of expression of collagen type X, a marker of hypertrophic chondrocytes was observed in all groups. It may be because of the TGF-β as previously reported (35).

## Conclusion

Our data indicate that PRP could promote the proliferation and chondrogenic differentiation of hADSCs into chondrocyte-like cells, in Transwell culture and these responses to PRP are dose dependent. It was found that 10% concentration of PRP is giving best results. Therefore, the optimal concentration of PRP should be determined for each individual cell type. Platelet-rich plasma contains unknown components that have beneficial effects on the proliferation and differentiation of ADSCs. For the clinical application of PRP, further studies using TGF- β1 in chondrogenic media are needed. 
